# Drug Delivery Applications of Hydrophobic Deep Eutectic Solvent-in-Water Nanoemulsions: A Comparative Analysis of Ultrasound Emulsification and Membrane-Assisted Nanoemulsification

**DOI:** 10.1021/acsami.4c13163

**Published:** 2024-12-31

**Authors:** Usman T. Syed, Javier Calzada, Gracia Mendoza, Manuel Arruebo, Emma Piacentini, Lidietta Giorno, João G. Crespo, Carla Brazinha, Victor Sebastian

**Affiliations:** † LAQV/Requimte, Department of Chemistry, NOVA School of Science and Technology, FCT NOVA, Universidade NOVA de Lisboa, 2829-516 Caparica, Portugal; ‡ Department of Chemical Engineering and Environmental Technology, Universidad de Zaragoza, Campus Río Ebro-Edificio I+D, 50018 Zaragoza, Spain; § Institute on Membrane Technology, National Research Council, ITM-CNR, via P. Bucci, 17/C, 87030 Rende, Cosenza, Italy; ∥ Department of Mechanical Engineering, ICAI School of Engineering, Universidad Pontificia Comillas, Alberto Aguilera, 25, 28015 Madrid, Spain; ⊥ Department of Pharmacology and Physiology, Forensic and Legal Medicine, Veterinary Faculty, University of Zaragoza, 50009 Zaragoza, Spain; # Aragon Health Research Institute (IIS Aragon), 50009 Zaragoza, Spain; ¶ Instituto de Nanociencia y Materiales de Aragón (INMA), Universidad de Zaragoza-CSIC, c/María de Luna 3, 50018 Zaragoza, Spain; ∇ Networking Research Center on Bioengineering, Biomaterials and Nanomedicine (CIBER-BBN), 28029 Madrid, Spain

**Keywords:** deep eutectic solvent, membrane emulsification, nanoemulsions, cytotoxicity, lidocaine

## Abstract

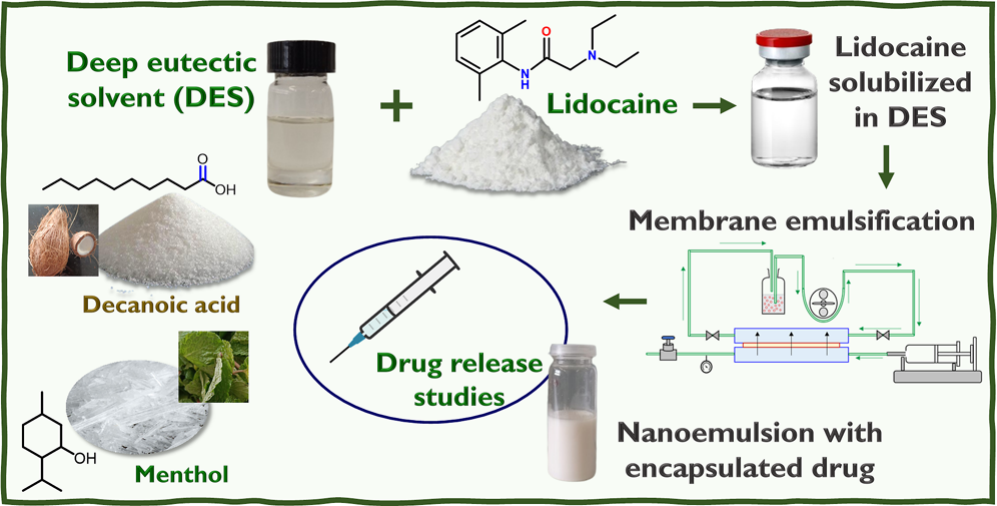

The emergence of green chemistry and engineering principles to enforce sustainability aspects has ensured the prevalence of green solvents and green processes. Our study addresses this quest by exploring drug delivery applications of hydrophobic deep eutectic solvents (DESs) which are alternative green solvents. Initially, this work showcases the hydrophobic drug solubilization capabilities of a natural hydrophobic DES, menthol, and decanoic acid. To consider biomedical applications wherein polar media are encountered, this work further demonstrates the potential drug delivery application of these systems by encapsulating the anti-inflammatory local anesthetic lidocaine in hydrophobic DES-in-water nanoemulsions. NMR studies confirm the high solubility of the hydrophobic drug in hydrophobic DES comprising menthol and decanoic acid (1:2 molar ratio). Ultrasound emulsification and energy-efficient membrane emulsification techniques were employed to disperse 4% (v/v) DES into a 2% (w/w) Tween 20 surfactant aqueous solution. An isoporous microengineered membrane (nominal pore size ∼ 9 μm) was used to produce lidocaine-loaded DES-based nanoemulsions. Such membrane-assisted nanoemulsification was possible because the hydrophobic DES exhibits relatively low interfacial tension with the continuous phase and acts as a cosurfactant. Moreover, increased concentrations of lidocaine within the DES resulted in a further decrease in the interfacial tension and a lower melting point. Among the kinetic models analyzed to evaluate the release of lidocaine encapsulated in hydrophobic DES-in-water nanoemulsions, the Korsmeyer–Peppas kinetic model provided the best fit. The release constant “*n*” of <0.5 indicates that the drug release mechanism is predominantly governed by diffusion. Additionally, cytotoxicity against various human cell lines demonstrated the nanoemulsion’s potential for anti-inflammatory drug delivery applications. Consequently, the nanoemulsion of DES presents a promising solution for the effective loading and delivery of poorly soluble drugs. This innovative approach enhances drug solubility and bioavailability, providing a versatile platform for controlled drug release. By leveraging the advantages of nanoemulsion technology, our study underscores the potential of DES-based formulations to promote drug delivery systems across a variety of therapeutic applications.

## 1 Introduction

Hydrophobic deep eutectic solvents (DESs) are a recent class of green solvents that offer 100% atom economy, biodegradability, chemical stability, low volatility, and nonflammability.^1^ The challenges attributed to traditional organic solvents paved the way for ionic liquids at the end of the last century in the pursuit of sustainability.^2^ Ionic liquids garnered attention as they possess unique physicochemical properties with negligible vapor pressure, thermal stability, intrinsic ability to dissolve various organic and inorganic compounds, and the flexibility to synthesize them as hydrophobic or hydrophilic solvents depending on the chosen ionic component.^3,4^ However, ionic liquid solvents raised several concerns when numerous studies reported them as toxic, exhibiting low biodegradability, and having unrelenting high purity requirements to sustain their physical properties. Also, for many of them, their high cost hinders their use at an industrial scale.^4,5^ This led to the development of alternative solvents termed (hydrophilic) DESs. Typically, DES is a liquid system formed from a eutectic mixture of Lewis or Brønsted acids and bases that results in a drop in the melting point of the solvent compared to its solid individual components. This class of solvents offers the tunability of ionic liquids but is economical, environmentally benign, and easy to be synthesized.^6^

At the beginning of this century, hydrophilic DESs were proposed to be applied in several sectors. However, in 2015, a separate class of DES was introduced and termed hydrophobic DES.^7,8^ Accordingly, researchers exploited the potential application of these analogues of hydrophobic ionic liquids. In the past few years, the increasing number of papers published in the field of hydrophobic DES has demonstrated applications mainly centered around extraction and other processes aiming at environmental protection.^1,9,10^ These range from extraction of biomolecules/platform chemicals/organic compounds, hydrometallurgy, microextraction of micropollutants, dyes or pigments, and as a medium for CO_2_ capture.^9,11−13^ Lately, the focus of these DESs shifted toward the chemical industry for biocatalysis, enantioseparation, material modification (such as ferrofluids and membranes), and energy production, storage, and conversion.^1,3,14−16^

The biomedical applications of hydrophobic DES are still scarce, although hydrophobic DESs have also exhibited biomedical prospects to match the therapeutic applications of their hydrophilic counterparts.^3,17−20^ An alternative biomedical application of therapeutic hydrophobic DES was explored by determining their antibacterial/antifungal properties. Increased antibacterial activity of hydrophobic DES in comparison that of their individual components was reported.^18,19^ However, the majority of biomedical applications require bioactive compounds to be soluble in a polar medium. To ensure this prerequisite, therapeutic hydrophobic DES emulsions might be dispersed in water and further stabilized by a surfactant. Hence, our group, using a membrane emulsification technique, sustainably produced hydrophobic DES-in-water nanoemulsions, and the antimicrobial results were compared with those provided by DESs prepared by the traditional ultrasound emulsification technique. The results obtained opened a new window for biomedical applications, as the nanoemulsions exhibited enhanced antimicrobial activity in comparison to the hydrophobic DES and its individual components.^19^ Later, a follow-up of this previous work annotated the role of the components of the therapeutic antibacterial hydrophobic DES-in-water nanoemulsions, wherein the designed solvents were synthesized by choosing the right component of hydrophobic DES to target a specific bacterial strain.^20^ More recently, a similar work of hydrophobic DES-in-water nanoemulsions by Zeng et al. demonstrated increased antibacterial activity against both Gram-positive and Gram-negative bacterial strains.^21^

Besides solubilizing active pharmaceutical ingredients by forming hydrophobic DESs, there is an enhancement in the hydrophobic drug solubilities for an array of hydrophilic DESs as new drug vehicles.^1,9,10^ Based on these reports, the present work aims at harnessing the possibility of enhanced drug loading using a low-cost and natural-based hydrophobic DES. This strategy could prove economical when compared with the approach of employing the given drug as a starting component to synthesize a hydrophobic DES for therapeutic purposes. Hence, our earlier work on the hydrophobic DES comprising solid compounds such as dl-menthol (hydrogen-bond acceptor) and decanoic acid (hydrogen-bond donor) in a 1:2 molar ratio and having an eutectic composition as the melting points of the solvent dipped to 14 °C^19^ was expanded in this work to solubilize poorly soluble hydrophobic drugs. The basis of using natural hydrophobic DES is based on recent reports wherein they are reported to have excellent solvation capacity.^22,23^ Subsequently, exploitation of the encapsulated drug in an emulsion medium for drug delivery applications sets the premise of the present study.

Nanoemulsions offer an increase in bioavailability of the active compounds, with their intrinsic high surface area for mass transfer, by loading them in the dispersed phase of the emulsion system.^24,25^ In comparison to traditional emulsification techniques including ultrasound emulsification, sustainable production of nanoemulsions has been demonstrated by membrane-based emulsification techniques as they offer advantages such as reduced energy costs, reduced surfactant concentration and facilitating complexity of surfactants’ composition, uniform emulsion droplets, mild operating conditions, and the possibility to scale up.^19,20,25^ In brief, membrane emulsification is a pressure-driven technique wherein the oil phase is pushed through the membrane pores and interfaced with the continuous phase (aqueous surfactant system) in the recirculation mode. The size of the pores usually controls the droplet size, and the detachment of the oil droplets from the membrane surface is achieved by the higher flow rate of the continuous phase.^25^ Recently, we introduced a subset of membrane emulsification techniques known as membrane-assisted nanoemulsification, wherein the size of the membrane pores rather assists and does not control the droplet size of emulsions. The process is controlled mostly by the physicochemical properties of the oily phases.^19,20^

In particular, in our previous work,^19^ we demonstrated the feasibility of membrane emulsification for the preparation of DES-in-water nanoemulsions, where DES was formed from dl-menthol and decanoic acid. In the present work, we exploited the ability of DES to solubilize hydrophobic drugs and the use of membrane emulsification to prepare drug-loaded hydrophobic DES nanoemulsions with high payload content and monomodal distribution. In addition, the kinetics of the release of the selected anti-inflammatory drug was investigated. Furthermore, to determine if these optimized nanoemulsions were suitable for biomedical purposes, cytotoxicity studies were performed on different cell lines (human dermal fibroblasts, human tumoral cells, human macrophages, and mouse mesenchymal stem cells (mMSCs)). Lastly, the novelty of this work involves initiating a new application of hydrophobic DESs by formulating drug-loaded hydrophobic DES-in-water nanoemulsions for drug delivery applications.

## 2 Materials and Methods

### 2.1 Materials

The microengineered membrane was fabricated by laser machining for which stainless steel AISI 304 foils of a thickness of 25 ± 2 μm (Record Metall-Folien GmbH Company, Germany) were used. A Q-switched diode-pumped laser from Rofin (model PowerLine S3 SHG, Germany) was used in a pulsed mode for the surface microperforation of the metallic membrane with 100 μm pitch (distance between 2 pores).

The two phases of the emulsions were selected as reported by Syed et al.,^19^ wherein the dispersed phase was a DES comprising dl-menthol (hydrogen-bond acceptor) and decanoic acid (hydrogen-bond donor) in a 1:2 molar ratio (98% and 95% purity, respectively, Sigma-Aldrich, Germany). Tween 20 (polysorbate 20, Sigma-Aldrich, Germany) at 2% (w/w) in water was used as the continuous phase. A Pur-A-Lyzer Maxi Dialysis Kit with dialysis tubes with 3.5 kDa molecular weight cutoff was purchased from Sigma-Aldrich, Germany. Five routinely used hydrophobic drugs, namely, benzocaine, ciprofloxacin, lidocaine, naproxen, and β-carotene (Sigma-Aldrich, Germany) were tested for drug solubility studies in the synthesized DES. Ethanol and DMSO were purchased from Panreac, Spain. Acetonitrile (ACN) was purchased from VWR (Avantor). Ultra-performance liquid chromatography (UPLC)-grade water was obtained from a Milli-Q Advantage A10 System with a resistivity of 18.2 mΩ (Merk Millipore, Germany). Milli-Q water was used for all of the experiments.

### 2.2 Hydrophobic Drug Solubilization Studies into the Synthesized Hydrophobic DES

Briefly, the DES components were heated at 85 °C and 350 rpm until a colorless liquid was formed. Eventually, the solvent was cooled to room temperature and stored at 25 °C until further use. The solubility of the aforementioned five hydrophobic drugs in DES was evaluated by adding 10 mg of these pure pharmaceutical ingredients in glass vials containing 1 g of DES. The vials were placed in an oil bath at 25 °C under continuous stirring for 12 h. When a homogeneous mixture was observed, indicating complete solubility of the drug in the DES, additional known quantities of the drugs were added until the mixture turned heterogeneous, which visually indicated the saturation point. The selected drug solubilized at 10% and 50% (w/v) in the hydrophobic DES was preserved at 37 °C for further emulsification studies (see Section S1 of the Supporting Information for the experimental procedure to synthesize and characterize the hydrophobic DES).

### 2.3 Characterization of the Drug-Loaded Hydrophobic DES to be Used as the Dispersed Phase

#### 2.3.1 Determination of Chemical Structure of Drug-Loaded DES by NMR Studies

Proton NMR studies were conducted by using a Bruker AV-400 spectrometer (Bruker BioSpin, USA) at 400 MHz using CDCl_3_ as a solvent to determine the chemical structure of the compounds. This study was performed to confirm the high solubility of the selected drug in the hydrophobic DES and to rule out the possibility of the formation of any new eutectic mixture.

#### 2.3.2 Determination of the Melting Point of Drug-Loaded DES by Differential Scanning Calorimetry Studies

Differential scanning calorimetry (DSC Q-200, TA Instruments, USA) was employed to analyze the solid–liquid phase transition of the dispersed phases (without and with drug-loaded DES).

#### 2.3.3 Interfacial Tension Measurements of the Liquid Phases Used

A Drop Shape Analyzer (DSA 25B, Kruss GmbH, Germany) was used to measure the interfacial tension between the drug-loaded dispersed phases and the continuous phase.

### 2.4 Ultrasound and Membrane Emulsification Studies to Produce Drug-Loaded DES-in-Water Nanoemulsions

The emulsification studies by ultrasound and membrane-based techniques were adapted from our earlier work.^19^ For producing small-sized DES-in-water nanoemulsions, an ultrasonic processor (Vibra Cell Sonics, VCX 505, USA) with 30% intensity and at 1 s pulse on/off mode was employed for 25 min to emulsify 2 mL of the dispersed phase comprising 4% (v/v) hydrophobic DES with and without 10% and 50% (w/v) of the selected hydrophobic drug in 50 mL of the continuous phase comprising 2% (w/w) Tween 20 aqueous solution. For a comparative study, DES-in-water nanoemulsions were also produced by a pressure-driven membrane emulsification. A microengineered isoporous stainless-steel membrane with a pore size of 9 μm and a membrane pitch (distance between 2 pores) of 100 μm was used. The dispersed phase was injected into the system by a syringe pump (Harvard Apparatus, PHD ULTRA 4400 I/W PROG, USA) and the continuous phase was recirculated via a peristaltic pump (Velp Scientific, SP311, Italy). The experimental procedure was followed as previously reported in ref (19) (see Section S2 of the Supporting Information for details on membrane emulsification studies).

### 2.5 Characterization of the Emulsions

The droplet size distribution and polydispersity index (PDI) of the emulsions were measured by dynamic light scattering using a NanoZetaSizer particle size analyzer (Malvern instruments, Nano ZS, UK) and were confirmed by undertaking TEM (FEI Tecnai T20 Company, USA) studies (see Section S3 of the Supporting Information for further experimental details).

### 2.6 In Vitro Drug Delivery Studies

The optimized hydrophobic DES-in-water nanoemulsions consisting of the encapsulated 10% and 50% (w/v) of the selected drug formulated by ultrasound and membrane emulsification techniques were subjected to in vitro drug delivery studies. One mL of the optimized nanoemulsion with the encapsulated drug was placed in a dialysis tube and submerged in a flat-bottomed falcon tube containing 40 mL deionized water along with a magnetic stirrer. This system was stirred at 350 rpm at 37 °C. At specific time intervals, one mL of the aqueous sample was collected and replaced with fresh deionized water. The samples collected were subjected to UPLC studies for the quantification of the drug release (see Section S4 of the Supporting Information for the experimental details).

The drug loading capacity was calculated based on the following equation, eq 1
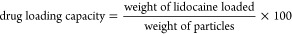
1

The drug encapsulation efficiency is correlated with the drug retention efficiency along time *t* during the drug release experiments. The retention efficiency at time *t* was calculated based on the following equation, eq 2
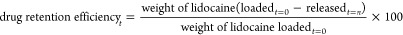
2

### 2.7 Cytotoxicity Studies

The synthesized DES and its individual components (dl-menthol and decanoic acid), the selected drug, and the optimized nanoemulsions without and with 50% (w/v) drug encapsulated in the hydrophobic DES were subjected to cytotoxicity studies for ensuring its suitability toward biomedical applications. The samples were filtered and dissolved in 90% (w/w) DMSO. These were tested against 4 cell lines, namely, human dermal fibroblasts, U251MG (human tumoral cell line derived from a glioblastoma), human macrophages, and mMSCs at varied concentrations. Fibroblasts and U251MG cells were cultured in DMEM high glucose (Biowest, France) containing 2 mM l-glutamine and supplemented with 10% v/v fetal bovine serum (Gibco, UK) and 1% penicillin–streptomycin–amphotericin B (Biowest, France). Human macrophages were obtained from monocytes cultured in RPMI 1640 (Biowest, France) containing 2 mM l-glutamine and supplemented with 10% v/v fetal bovine serum (Gibco, UK), 1% HEPES (Lonza, Belgium), 0.1% 2-mercaptoethanol (50 mM) (Gibco, UK), 1% nonessential amino acids, 1% sodium pyruvate (100 mM), and 1% penicillin–streptomycin–amphotericin B (Biowest, France). Macrophages were obtained by the in vitro differentiation of monocytes by means of the addition of 1 μM phorbol 12-myristate 13-acetate (PMA) (Sigma-Aldrich, US) to the cell medium for 72 h. Finally, mMSCs were grown in DMEM-F12 (Biowest, France) supplemented with 1% glutamine (Gibco, UK), 10% v/v fetal bovine serum (Gibco, UK), and 1% penicillin–streptomycin–amphotericin B (Biowest, France). All cell lines were cultured with 5% CO_2_ at 37 °C except for mMSCs which were cultured under hypoxic conditions (3% O_2_).

The blue cell viability assay (Abnova, Taiwan) was carried out to evaluate the viability related to cell metabolism after incubation with the nanoemulsion and its components for 24 h. The reagent was added to the cells (10%) and incubated for 4 h at 37 °C. The fluorescence was then recorded in a microplate reader (535/590 nm ex/em; Synergy HT, BioTek, USA). Control samples without the cells were also analyzed to assess whether the nanoemulsion or its components would interfere with the assays. Cell viability was determined by the interpolation of the emission data obtained from the treated samples and the control samples (control samples = 100% viability).

## 3 Results and Discussion

### 3.1 Screening Studies for Evaluating the Solubility of Various Hydrophobic Drugs into the DES

Although there are several studies reporting the use of hydrophobic DESs in (micro)extraction and environmental applications, their use in biomedical applications is not yet widely exploited.^1,9,10^ This study evaluates the usage of hydrophobic drugs that can be encapsulated within the DES-in-water nanoemulsions, whereby the specific surface area and loading of the drugs can be enhanced to promote increased bioavailability.

As a starting point, five different hydrophobic anti-inflammatory drugs were tested for their solubility into the hydrophobic DES. As seen in Table 1, β-carotene, benzocaine, and naproxen drugs are insufficiently soluble in the menthol and decanoic acid-based hydrophobic DES. Ciprofloxacin showed marginal solubility into the DES. However, the solubility of lidocaine into the hydrophobic DES was significant as more than 750 mg of the drug was easily solubilized in 1 g of DES. This is at least 15 times higher than the solubility results obtained when harsh solvents such as DMSO or DMF are used.

**Table 1:** Solubility of Various Hydrophobic Drugs in Water, Organic Solvents,^26^ and Comparison with the DES under Study at 25 °C

hydrophobic drug	solubility in water at 25 °C [mg·mL^–1^]	solubility in solvents	solubility in DES under study [mg·g^–1^ of DES]
ciprofloxacin	insoluble; but at pH 4–5, soluble ≈40	insoluble in ethanol and poorly soluble in DMSO	22.18
naproxen	0.016	slightly soluble in ether; soluble in methanol, chloroform	13.31
benzocaine	1.31	500 mg·mL^–1^ in methanol and chloroform, 250 mg·mL^–1^ in ether, 50 mg·mL^–1^ in ethanol	47.0
β-carotene	insoluble	4.5 mg·g^–1^ in dichloromethane, 0.1 mg·mL^–1^ in hexane	1.25
lidocaine	**4.1**	**50 mg·mL**^**–1**^ in ethanol, **47 mg·mL**^**–1**^**in methanol and DMSO**	**>750**

A review by van Osch et al. compiled a list of hydrophobic DES synthesized using lidocaine as the hydrogen-bond acceptor. These included menthol, thymol, decanoic acid, or dodecanoic acid.^17^ For synthesizing any DES, two individual hydrophobic compounds must be stirred in a specific molar ratio at temperatures higher than the melting point of one (or both) of the individual compounds. In this study, once the hydrophobic DES was synthesized, the lidocaine drug having a melting point of 68 °C^26^ was solubilized by adding and stirring in the already synthesized DES at room temperature without any additional heat supply. This drug solubilization strategy is efficient and cost-effective as the drug can be loaded and customized in higher quantities with increased energy savings and without having the risk of destabilization of the DES or unsuccessful formation of the DES as only specific molar ratios of the lidocaine are successful for the formation of DES having varied hydrogen-bond donors.^17^ Thus, the DES systems with and without the lidocaine drug solubilized were further subjected to characterization studies.

### 3.2 Characterization of the Dispersed Phase: Hydrophobic DES with the Solubilized Lidocaine Drug

#### 3.2.1 NMR Spectroscopy Studies

**Figure 1:**
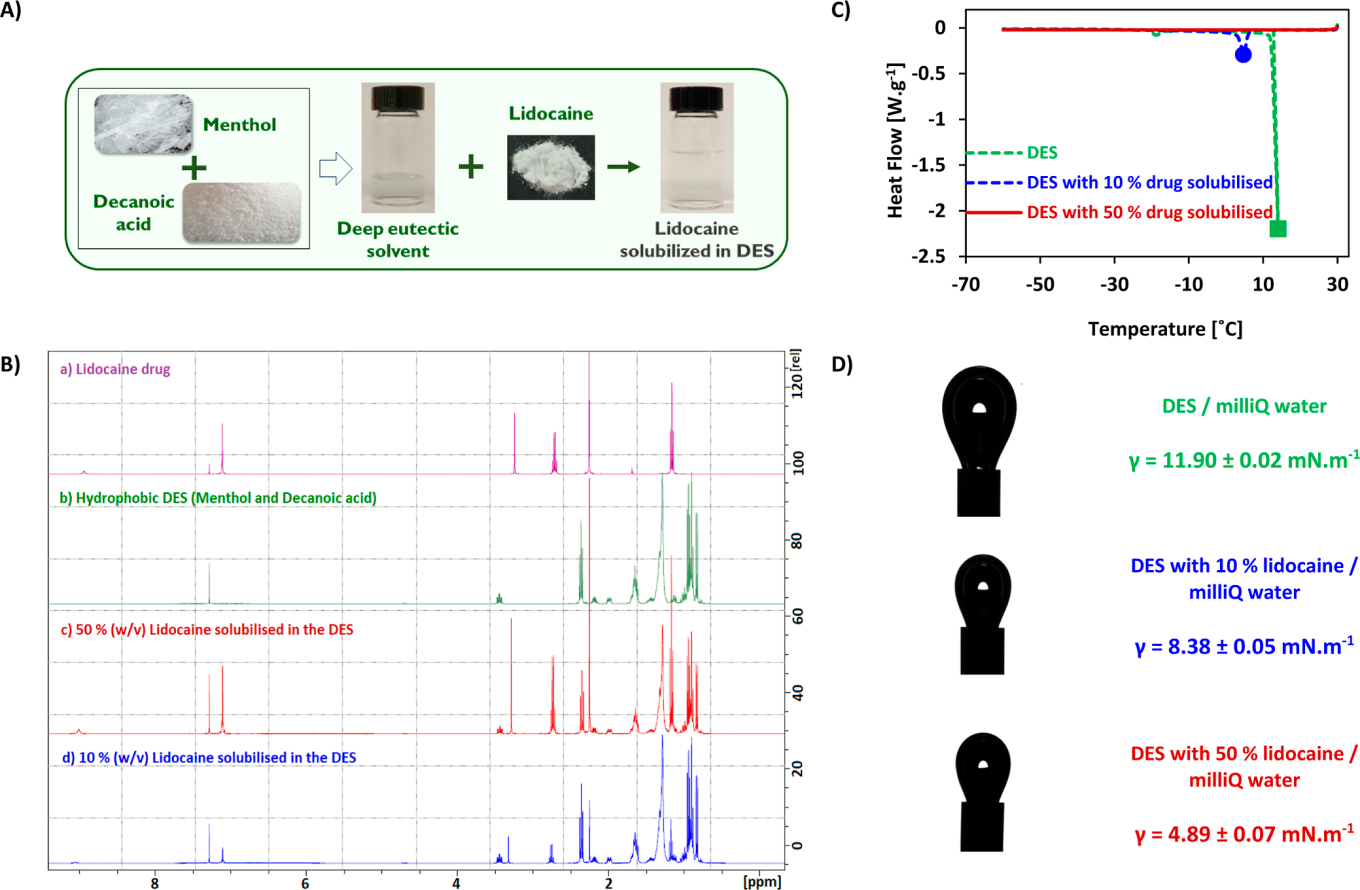
Characterization of the dispersed phases to be used for emulsification studies: (A) digital images of the lidocaine drug solubilized in the DES, (B) NMR studies, (C) DSC studies, and (D) interfacial studies of hydrophobic DES, 10% (w/v) lidocaine solubilized in the DES and 50% (w/v) lidocaine solubilized in the DES.

Proton nuclear magnetic resonance (^1^H NMR) spectroscopy studies were carried out to determine the chemical structure of the compounds, to confirm the high solubility of lidocaine in the hydrophobic DES and to rule out the possibility of the formation of any new eutectic mixture (see Figure 1A).

The individual ^1^H NMR peaks of pure compounds (dl-menthol and decanoic acid) that comprise the hydrophobic DES can be viewed in the PubChem database.^26^ However, as reported by numerous studies, when two components are mixed together to form eutectic mixtures, the structure of the resultant molecules in the DES changes.^5,7^ This is evident in the NMR spectrum of the hydrophobic DES as seen in Figure 1. Moreover, when the hydrophobic lidocaine drug (white solid powder) was solubilized at different concentrations in the hydrophobic DES by overnight stirring, it resulted in a clear transparent liquid. These samples along with pure lidocaine were also subjected to NMR studies. The complete solubilization of lidocaine in the DES is seen in Figure 1A. Furthermore, Figure 1B reveals that the NMR spectra of the lidocaine-solubilized hydrophobic DESs are a mere overlap of the NMR spectrum of pure lidocaine and unmodified hydrophobic DES. These results confirm that the lidocaine drug does not form a eutectic mixture with menthol and decanoic acid but is prone to high solubility in the hydrophobic DES formed by dl-menthol and decanoic acid. The individual NMR spectra are presented in Figures S1–S4. Thus, these results cement our working strategy to encapsulate lidocaine in hydrophobic DES-in-water nanoemulsions.

#### 3.2.2 DSC Studies

DSC was employed to determine the phase transition of the dispersed phases along the temperature profile. Figure 1B illustrates the melting points of the 3 different dispersed phases used in the present study that include hydrophobic DES, 10% (w/v) lidocaine solubilized in the DES, and 50% (w/v) lidocaine solubilized in the DES.

The hydrophobic DES comprises dl-menthol and decanoic acid having individual melting points of 36 and 31 °C, respectively.^19^ However, when these components were combined to synthesize their eutectic mixture, a resultant decrease in the melting point was observed. As seen in Figure 1C, a clear solution of the hydrophobic DES has a melting point of around 11–14 °C. Further reduction of the temperature resulted in a gel-like behavior with increased viscosity. The small dip at −20 °C could possibly indicate the glass transition *T*_g_ temperature. Furthermore, when lidocaine was solubilized at 10% (w/v) in the DES, there was a shift in the solid–liquid phase transition profile. Instead of the DES melting above 10 °C, the presence of the hydrophobic drug lowered the melting point of the eutectic solvent to 3.5–5.5 °C. Lastly, when the amount of the lidocaine drug increased to 50% (w/v) in the DES, the melting point reduced further and, as seen in Figure 1B, the eutectic solvent did not melt until −60 °C. It could be hypothesized that the positively charged lidocaine molecule provides nonsymmetric ions to the decanoic acid with low lattice energy and further decreases the melting point of the dispersed phase.^27,28^ Thus, all 3 types of dispersed phases were deemed to be suitable for performing ultrasound and membrane emulsification studies at laboratory room temperature conditions.

#### 3.2.3 Interfacial Tension Studies

The pendant drop method was employed to measure the interfacial tension between the aqueous phase and the hydrophobic DES with or without the solubilized lidocaine. Figure 1C depicts the interfacial tension measurements of the various solutions in contact with water. The formation of emulsions can be predicted by the interfacial tension between the two immiscible phases. As seen in Figure 1D, it is interesting to note the reduced interfacial tension between the hydrophobic DES and Milli-Q water, considering that they are immiscible.

Usually, for those dispersed-phase solutions that have high interfacial tension with the continuous phase, the type and concentration of the surfactant play a prominent role in forming smaller-sized emulsion droplets with enhanced kinetic stability.^24,25^ For the formulation of oil-in-water emulsions by membrane emulsification, typical commercial oils or perfluorocarbons have been studied exhibiting interfacial tension with water in the range of 22–26 mN·m^–1^ and 53–70 mN·m^–1^, respectively.^24^

On the other hand, it is interesting to note that the present study shows a low interfacial tension between the hydrophobic DES and water. This is in accordance with a recent study by Syed et al., wherein an interfacial tension of ∼8 mN·m^–1^ was witnessed between water and a DES system (comprising therapeutic terpenes such as menthol and thymol).^20^ Although the hydrophobic DES has a negligible solubility in water, its interfacial tension with water is surprisingly low. This could be advantageous, as conventional surfactants with high hydrophilic–lipophilic balance (HLB) values can be incorporated at low concentrations in oil-in-water emulsification studies. Furthermore, with the increase in the concentration of the hydrophobic anti-inflammatory lidocaine in the DES, a decrease in the interfacial tension values between the dispersed phase and water was observed. This could prove to be beneficial for emulsification studies. Lastly, this specific combination of DES has been well studied in terms of its viscosity, acidity, and conductivity.^19,29,30^

### 3.3 Emulsification Studies to Produce Lidocaine-Loaded DES-in-Water Nanoemulsions

**Figure 2:**
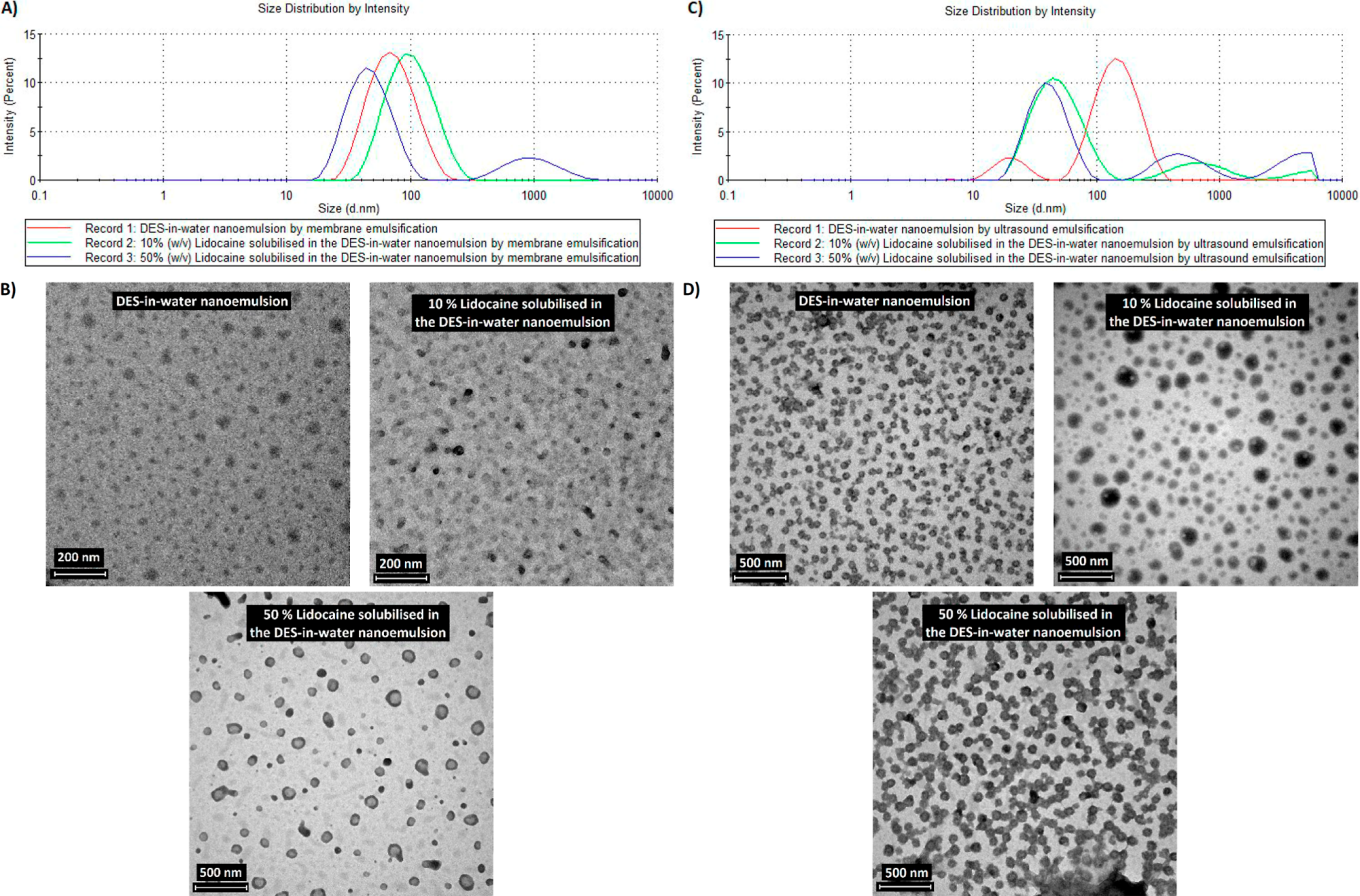
Characterization of the hydrophobic DES-in-water nanoemulsions: (A) DLS and (B) TEM images of membrane emulsification studies. (C) DLS and (D) TEM images of ultrasound emulsification studies for varied dispersed phases with and without the loading of lidocaine.

The primary objective of this study was to encapsulate the selected hydrophobic anti-inflammatory drug in the DES and disperse it in nanoemulsions for potential biomedical applications. Two different emulsification techniques were used: traditional ultrasound emulsification and membrane emulsification. The operating conditions and the ratio of the dispersed phase and continuous phase were kept constant as reported in our earlier study.^19^ The membrane emulsification unit employed in this work is depicted in Figure S5. For membrane emulsification studies, the maximum cross-flow velocity of the continuous phase, ν_c_ of 0.32 m·s^–1^, that can be attained in the present emulsification setup was used as higher cross-flow velocity corresponds to higher wall shear stress. This facilitates the droplet detachment from the membrane surface along with reduced emulsion droplet sizes.^25,31,32^Figure 2 depicts the characterization results of the optimized nanoemulsions.

As seen in Figure 2A, a monomodal distribution of hydrophobic DES-in-water nanoemulsions was obtained having a *Z*_avg_ mean droplet size of 58.7 ± 0.4 nm and a PDI of 0.21. Changes in the DLS profiles were observed when the dispersed phase was modified by solubilizing lidocaine at 10% and 50% (w/v). With the inclusion of 10% (w/v) of lidocaine, the drug-encapsulated DES-in-water nanoemulsions exhibited emulsion droplet sizes of 91.1 ± 0.3 nm and a PDI of 0.17. However, when the concentration of the drug was increased to 50% (w/v), a bimodal distribution of the emulsion profile was seen with the majority of the emulsions centered at around 50 nm and a significant fraction at 800 nm indicating that part of lidocaine was not encapsulated in the bulk of the emulsion droplets. When lidocaine was included, the emulsion droplets demonstrated altered size distributions, reflecting the interaction between the drug and the hydrophobic DES. Although specific values for drug loading and encapsulation efficiency were not accurately determined, the overall behavior of the nanoemulsions implies effective retention of lidocaine, facilitated by the unique properties of the hydrophobic DES, which acts as a barrier to prevent rapid drug release.

The DLS profiles of the nanoemulsions, measured immediately and after 4 weeks (while being preserved at 30 °C), both with and without lidocaine loaded, are depicted in Figure S6. Notably, there is a minimal change in the size of the emulsions over this period, indicating good long-term storage stability. This suggests that the nanoemulsions are not significantly affected by Ostwald ripening under the conditions tested.

The size and size distribution of the optimized nanoemulsions measured by DLS for the membrane emulsification studies were complemented by TEM measurements as depicted in Figure 2B. Moreover, for all three dispersed phases, better results in terms of lower *Z*_avg_ and lower PDI of the emulsions were achieved at operating conditions of 0.02 mL·min^–1^ as the *Q*_dp_ dispersed-phase flow rate when compared with a *Q*_dp_ of 0.2 mL·min^–1^. These results are in accordance with the studies reported in the literature which portray that the lower dispersed-phase flux results in a reduced size of the resulting emulsions with controlled detachment from the membrane surface.^19,20^ Furthermore, in a direct membrane emulsification process, the size of the pores controls the size of the emulsions. However, the present study was accomplished with an isoporous microengineered membrane that was fabricated with 9 μm pores on the active (top) surface by a laser drilling technique. This technique has limitations as it cannot produce metallic membranes of isopores of less than 9 μm in diameter.^19^ However, as seen from the results presented in Figure 2A,B, the pore size of the membrane does not control the size of the emulsions formed as ∼60 nm monomodally distributed nanoemulsions with low polydispersity were formulated with this membrane. Therefore, as reported in our earlier work, this process cannot be classified as a direct membrane emulsification technique but rather as a “*membrane-assisted nanoemulsification*” technique wherein the membrane assists rather than controls the size of the resulting emulsions.^19,20,33^ A plausible explanation for this could be the self-assembly traits of the hydrophobic DES when in contact with the aqueous surfactant solution and the reduced interfacial tension of the hydrophobic DES with water (Figure 1C). This indicates that the hydrophobic DES itself possibly acts as a cosurfactant in the drug-loaded hydrophobic DES-in-water nanoemulsion. Furthermore, the self-assembly of surfactant systems in DES for drug delivery applications has been documented by Basu et al.^34^ The amphiphilic self-assembled phases arise from the solvent’s polarity and the solvophobic interactions between the hydrophobic tail groups of the amphiphiles.^35^ Notably, DES can act as surfactants or cosurfactants, reducing interfacial tension between the two phases and lowering the critical micelle concentration of the surfactant.^36,37^ For additional details on the effect of dispersed-phase flow rates on emulsion size and dispersity, please refer to Table S1 in the Supporting Information.

As depicted by the results of ultrasound emulsification in Figure 2C,D, the majority of the emulsion droplet sizes are in the nanoscale range. Moreover, the DES-in-water nanoemulsions without lidocaine produced by ultrasound emulsification seem to be polydispersed, as a monomodal distribution was not achieved. The emulsions become more polydisperse with an increase of lidocaine in them. As expected, the results achieved with a drug-containing dispersed phase in DES confirm that membrane emulsification provides better control over the dispersity of the emulsion nanodroplets than ultrasound emulsification, as previously observed when DES alone was used as the dispersed phase.^19,20^ Lastly, the nanoemulsions formulated by ultrasound emulsification and membrane emulsification at a dispersed-phase flow rate of 0.02 mL·min^–1^ with two different dispersed phases, (a) 10% (w/v) lidocaine solubilized in the DES and (b) 50% (w/v) lidocaine solubilized in the DES, were selected for the drug delivery studies.

### 3.4 Drug Delivery Experiments of the Selected Emulsions

From the application perspective, these optimized DES-in-water nanoemulsions are intended to be used as drug delivery carriers of the anti-inflammatory local anesthetic. Dialysis tubes were employed to carry out the in vitro drug release studies under sink conditions. UPLC studies were performed to determine the amount of selected anti-inflammatory lidocaine released from the DES-in-water nanoemulsions at specific time intervals. The drug-loading capacity of lidocaine was calculated based on eq 1. For the experiments of 10% (w/v) of lidocaine solubilized in the DES, the drug-loading capacity was 7.1% wherein the weight of particles was attributed to the amount of lidocaine, DES, and Tween 20 surfactant used to formulate the nanoemulsions. Similarly, for 50% (w/v) of lidocaine solubilized in the DES, the drug-loading capacity was 34.5%. From the UPLC data collected, the retention efficiency of the drug was calculated based on eq 2. For the 10% and 50% of the lidocaine solubilized in DES-based nanoemulsions by membrane emulsification, the retention efficiency at the initial 5 min of the drug release experiments was found to be 99.4% and 98.7%. For the ultrasound-based counterparts, it was 98.4% and 98.2%. Since the drug encapsulation efficiency can be directly correlated to drug retention efficiency, it can be safely concluded that for all nanoemulsions formulated by both techniques, the drug encapsulation efficiency is greater than 98%. Furthermore, four different routinely applied models were used to evaluate the kinetics of drug release.^31^Table 2 compiles the rate constants and *R*^2^ values obtained by the zero-order kinetic model, the first-order kinetic model, the Higuchi kinetic model, and the Korsmeyer–Peppas kinetic model for evaluating the kinetics of lidocaine release from DES-in-water nanoemulsions (see Table S2 for further details about the kinetic models studied).

**Table 2:** Rate Constants and *R*^2^ Values Obtained by Various Models to Evaluate the Kinetics of Lidocaine Release from DES-in-Water Nanoemulsion

kinetic model	% drug loaded in the DES-in-water nanoemulsion	emulsification method	release constant (*K*)	release exponent (*n*)	regression coefficient (*R*^2^)
zero order	10	membrane	38.50		0.37
		ultrasound	22.72		0.57
	50	membrane	13.67		0.47
		ultrasound	12.60		0.39
first order	10	membrane	26.35		0.19
		ultrasound	12.93		0.35
	50	membrane	10.44		0.32
		ultrasound	8.81		0.29
Higuchi	10	membrane	10.99		0.63
		ultrasound	15.45		0.87
	50	membrane	5.02		0.75
		ultrasound	5.51		0.71
Korsmeyer–Peppas	10	membrane	0.35	0.32	0.67
		ultrasound	0.22	0.48	0.96
	50	membrane	0.14	0.29	0.89
		ultrasound	0.13	0.35	0.92

As seen in Table 2, *R*^2^ values indicate that the classical kinetic models such as zero-order and first-order models are not a good fit for the employed case study of lidocaine encapsulation in the DES and its subsequent release from the DES-in-water nanoemulsions. These two models consider a constant or linear increase of a release component over a specified duration of time and their profiles are depicted in Figure S3 of the Supporting Information. Additionally, two other kinetic models, such as the Higuchi and the Korsmeyer–Peppas models, were studied.

**Figure 3:**
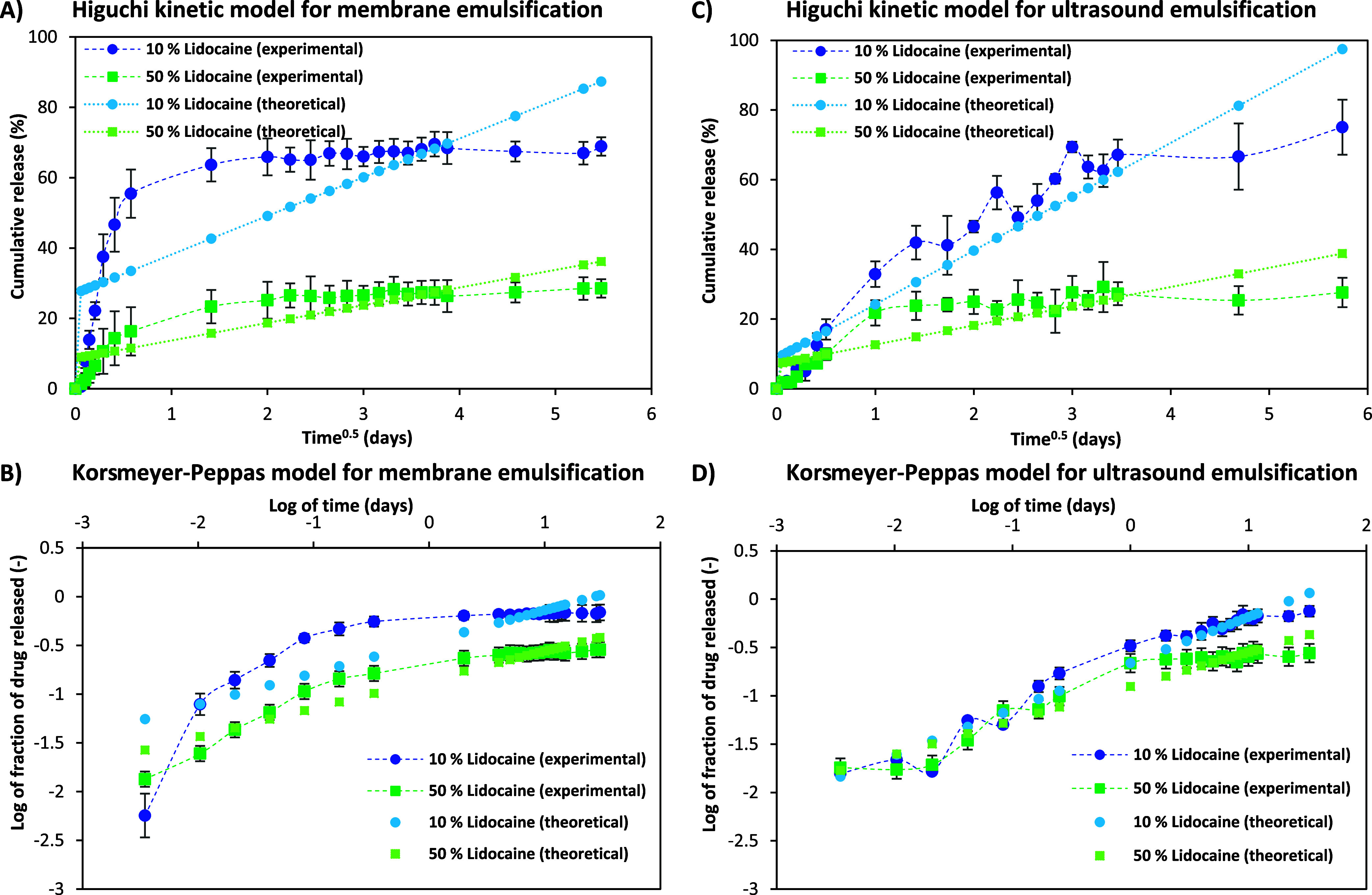
Kinetic release studies of lidocaine drug loaded in DES-in-water nanoemulsions produced by two emulsification techniques wherein UPLC measurements were done in triplicates. Membrane emulsification: (A) Higuchi kinetic model and (B) Korsmeyer–Peppas kinetic model. Ultrasound emulsification: (C) Higuchi kinetic model and (D) Korsmeyer–Peppas kinetic model.

Figure 3 depicts the profiles of Higuchi and Korsmeyer–Peppas kinetic models used to evaluate the lidocaine release from the DES-in-water nanoemulsions produced by membrane and ultrasound emulsification techniques. The Higuchi model was established to study the release of a solute-like drug as a square root of the time-dependent process, based on the Fickian diffusion equation.^38^ As seen in Figure 3A,B, there is a difference in the release profile of the membrane and ultrasound-based nanoemulsions that had 10% (w/v) and 50% (w/v) of the lidocaine loaded in the DES-in-water emulsion system. For both lidocaine concentrations, within 12 h, 60–80% of the drug was released and it reached stability in 2 days when the nanoemulsions were formulated by the membrane-assisted nanoemulsification technique. Comparatively, it took 1 day longer for the ultrasound-based nanoemulsions to achieve similar cumulative release values.

Alternatively, the Korsmeyer–Peppas kinetic model seems to better explain the mechanism of drug release for all of the systems studied. Table 2 also indicates that this model exhibited better fitting when compared with the others in terms of *R*^2^ values. Formerly, the Korsmeyer–Peppas kinetic model was introduced as a comprehensive semiempirical method to simultaneously explain the drug release due to the diffusion of water into the matrix, its swelling, and dissolution.^39^ As seen in Figure 3C,D, there seems to be an initial burst of lidocaine from the DES-in-water nanoemulsions into the hydrophilic compartment. The intensity of the instant release seems to be more prominent for the nanoemulsions obtained by membrane emulsification when compared to the ultrasound emulsification ones. Additionally, as seen in Table 2, the release constant “*n*”values are lower for the emulsions produced by the membrane-based process. This could be because membrane emulsification leads to a better controlled size and dispersity of the emulsion droplets. According to Permanadewi et al., the spherical encapsulation of a drug shows a Fickian diffusion release mechanism, and this seems to be more prominent for both 10% (w/v) and 50% (w/v) drug loaded into the nanoemulsions produced by membrane emulsification and for the 10% (w/v) lidocaine drug loaded into the nanoemulsions produced by ultrasound emulsification.^40^ A plausible explanation for the “*n*” value to be the highest for the 50% (w/v) drug loaded into the ultrasound-based process to produce emulsions could be the inability of the ultrasound emulsification process to produce emulsions with smaller droplet sizes. Moreover, all four systems subjected to the kinetic study have release constant “*n*” values < 0.5. This suggests that the mechanism of drug release is governed by the diffusion process.^41^

### 3.5 Cytotoxicity Studies Using Human and Murine Cell Lines

**Figure 4:**
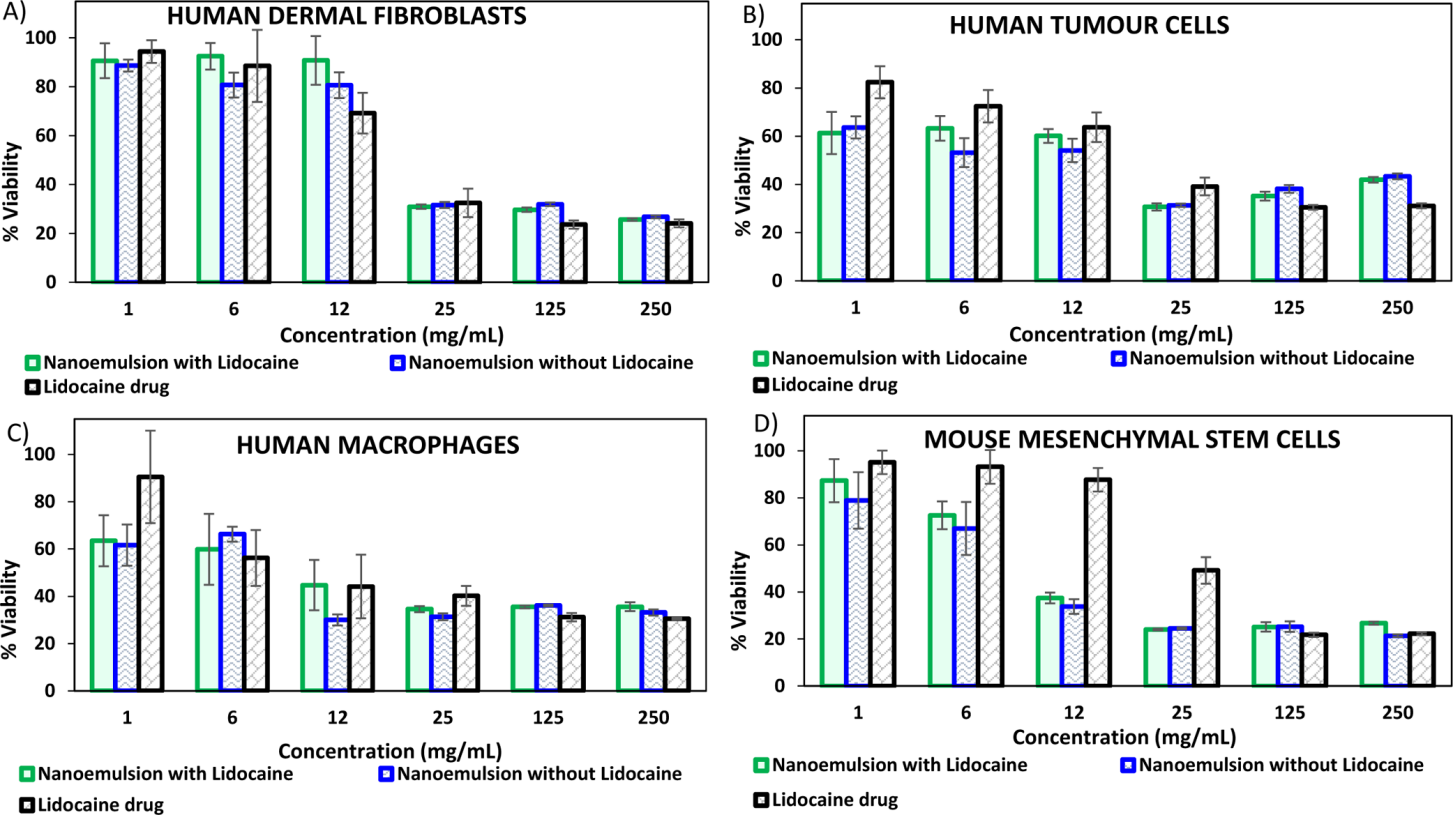
Cytotoxicity studies of the free lidocaine drug and optimized nanoemulsions with and without the 50% (w/v) lidocaine drug against: (A) human dermal fibroblasts, (B) human tumor cells, (C) human macrophages, and (D) mMSCs.

Since these optimized DES-in-water nanoemulsions are targeted for biomedical applications, it becomes imperative to determine the cytotoxicity of these nanoemulsions. As seen in Figure 4, the optimized nanoemulsions (with and without the lidocaine in the dispersed phase) were tested up to 250 mg·mL^–1^ against the four cell lines. The concentrations of the lidocaine drug tested were in the range of 0.01–2 mg·mL^–1^ that corresponded to the amount of lidocaine loaded in the nanoemulsions. Other samples such as individual components of the DES (dl-menthol and decanoic acid) and the synthesized DES were also tested against these four cell lines. The results were in accordance with the literature wherein the menthol-based natural DESs were cytocompatible with human and murine cell lines.^42−44^ However, due to their irrelevance in the final product aimed for biomedical use, they are not included in the results represented in Figure 4. As per the guidelines of ISO 10993-5 (Biological evaluation of medical devices: Tests for in vitro cytotoxicity), any sample is considered as cytotoxic when the corresponding cell viability is reduced by more than 30%.^45^ Accordingly, it can be safely deduced that the concentrations of the nanoemulsions that encapsulate the lidocaine drug are not cytotoxic to human dermal fibroblasts even up to 12 mg·mL^–1^ and for mMSCs up to 6 mg·mL^–1^. However, the nanoemulsions are cytocompatible with human tumoral cells and human macrophages only below 1 mg·mL^–1^. It can be deduced that nanoemulsions can be differently susceptible to different cell lines. Overall, the cytocompatibility results of the lidocaine drug loaded in DES-in-water nanoemulsions with various cell lines broaden the window of therapeutic applications for hydrophobic DES.

## 4 Conclusions

A detailed research strategy was implemented to formulate drug-encapsulated DES-in-water nanoemulsions and investigate their drug delivery behavior to expand the use of hydrophobic DES in biomedical applications. To achieve this, a well-studied hydrophobic DES comprising cheap and natural components such as menthol and decanoic acid in the molar ratio of 1:2 was selected, and it was successfully able to load a large quantity of a poorly soluble anti-inflammatory local anesthetic (i.e., lidocaine). To confirm the solubilization of the drug and that multiple eutectic mixtures are not formed, NMR studies helped to assess the chemical structure of the hydrophobic DES with and without the selected drug solubilized in it.

This study also demonstrated the benefits of using a membrane-assisted nanoemulsification process, as the 9 μm isoporous microengineered membrane enabled the formulation of hydrophobic DES-in-water nanoemulsions at the nanoscale range. A plausible explanation for the formation of emulsion droplets at the nanolevel while employing a membrane with microlevel pores was attributed to the self-assembly of the hydrophobic DES (with and without the lidocaine drug) within the emulsion system due to its unique physicochemical properties. Furthermore, the formulation of nanoemulsions of DES containing the lidocaine drug enhanced the solubility of the drug and its bioavailability as it offers inherent high specific surface area for mass transfer, rendering a promising solution for the delivery of drugs which are known to be poorly soluble in aqueous medium.

The drug delivery studies performed using dialysis tubes demonstrated that the lidocaine-loaded DES-in-water nanoemulsions produced by membrane emulsification had a more prominent initial burst of the drug from the matrix than the one shown by the emulsions prepared by ultrasound emulsification. These observations were supported by mathematical modeling studies. Additionally, the validation of the cytocompatibility of the hydrophobic DES-in-water nanoemulsions with various human cell lines at varied concentrations ensures their implementation for potential drug delivery applications. In future studies, various relevant anticancer, antituberculosis, or antiviral hydrophobic drugs could be tested for drug encapsulation and release. Lastly, our study successfully demonstrated a platform wherein the advantages of DES and nanoemulsion technology were amalgamated for effective loading and controlled drug release, thereby transforming drug delivery systems for a wide range of therapeutic applications.

## Supporting Information Available

Synthesis and characterization of hydrophobic DES used as the dispersed phase; NMR studies of the dispersed phase with and without the lidocaine drug loaded; NMR spectra of the DES comprising menthol and decanoic acid at a 1:2 molar ratio; NMR spectra of the pure lidocaine drug; NMR spectra with peaks of interest for 10% (w/v) lidocaine solubilized in the DES; NMR spectra with peaks of interest for 50% (w/v) lidocaine solubilized in the DES; interfacial tension measurements of the liquid phases used; experimental protocols for the membrane emulsification process, including conditions such as flow rate, pressure, and membrane properties; schematic representation of the membrane emulsification setup used in this study; comprehensive data on the characterization of the emulsions, including droplet size distribution, stability analysis, and viscosity measurements; summary of membrane emulsification experiments with relevant parameters and outcomes; characterization of the long-term storage of nanoemulsions without and with the lidocaine encapsulated; methodology and results from UPLC analysis to determine the release profile of lidocaine from the DES-in-water nanoemulsions; kinetic release studies of lidocaine drug loaded in DES-in-water nanoemulsions produced by two emulsification techniques; and kinetic models used to analyze the release kinetics of the drug, including parameters such as rate constants and fit quality for different models (PDF)

## Abbreviations

DES: deep eutectic solvent
DLS: dynamic light scattering
DSC: differential scanning calorimetry
HLB: hydrophilic–lipophilic balance
MANE: membrane-assisted nanoemulsification
NMR: nuclear magnetic resonance
PDI: polydispersity index
SEM: scanning electron microscopy
SPG: Shirasu porous glass
TEM: transmission electron microscopy
α: amplitude of the continuous phase flow [m]
ν_c_: cross-flow velocity of the continuous phase [m·s^–1^]
ρ_c_: density of the continuous phase [kg·m^–3^]
ρ_d_: density of the dispersed phase [kg·m^–3^]
τ_W_: continuous phase wall shear stress [Pa]
μ_c_: viscosity of the continuous phase [Pa·s]
*A*_mem_: active membrane surface area [m^2^]
*f*: frequency of oscillation of the continuous phase flow [Hz]
*M*_d_: dispersed-phase mass flow rate [kg·s^–1^]
*Q*_CP_: continuous-phase flow rate [m^3^·s^–1^]
*Q*_DP_: dispersed-phase flow rate [m^3^·s^–1^]
*V*_c_: volume of the continuous phase [mL]
*Z*_avg_: mean droplet diameter [nm]
