# Supplementary material for: Drug Delivery Applications of Hydrophobic Deep Eutectic Solvent-in-Water Nanoemulsions: A Comparative Analysis of Ultrasound Emulsification and Membrane-Assisted Nanoemulsification
Source: ACS Appl Mater Interfaces. 2024 Dec 31;17(2):4075–86. doi: 10.1021/acsami.4c13163 (PMC11744506; doi:10.1021/acsami.4c13163)
Supplement: Supplementary file 1 — am4c13163_si_001.pdf [file am4c13163_si_001.pdf]

Supporting Information

Drug delivery applications of hydrophobic deep eutectic solvent-in-water nanoemulsions: a comparative analysis of ultrasound emulsification and membrane-assisted nanoemulsification

Usman T. Syed [a,b,c], Javier Calzada [d], Gracia Mendoza [e,f], Manuel Arruebo [b,f,g], Emma Piacentini [c], Lidietta Giorno [c], João G. Crespo [a], Carla Brazinha [a*], Victor Sebastian [b,f,g,h*]

[a] LAQV/Requimte, Department of Chemistry, NOVA School of Science and Technology, FCT NOVA, Universidade NOVA de Lisboa, 2829-516 Caparica, Portugal
[b] Department of Chemical Engineering and Environmental Technology, Universidad de Zaragoza, Campus Río Ebro-Edificio I+D, 50018 Zaragoza, Spain
[c] Institute on Membrane Technology, National Research Council, ITM-CNR, via P. Bucci, 17/C, 87030, Rende, Cosenza, Italy
[d] Department of Mechanical Engineering, ICAI School of Engineering. Universidad Pontificia Comillas, Alberto Aguilera, 25, 28015 Madrid, Spain
[e] Department of Pharmacology and Physiology, Forensic and Legal Medicine, Veterinary Faculty, University of Zaragoza, 50009 Zaragoza, Spain
[f] Aragon Health Research Institute (IIS Aragon), 50009-Zaragoza, Spain
[g] Instituto de Nanociencia y Materiales de Aragón (INMA), Universidad de Zaragoza-CSIC, c/ María de Luna 3, 50018 Zaragoza, Spain
[h] Networking Research Center on Bioengineering Biomaterials and Nanomedicine (CIBER- BBN), Madrid, Spain

***Corresponding Authors:**

***** Victor Sebastian: victorse@unizar.es

* Carla Brazinha: c.brazinha@fct.unl.pt

Supporting information contains the following:

Number of Pages: 9

Number of Figures: 7

Number of Tables: 2

S1. Synthesis and characterisation of hydrophobic DES used as dispersed phase

The hydrophobic deep eutectic solvent is constituted of 1:2 molar ratio of DL-menthol (hydrogen bond acceptor) and decanoic acid (hydrogen bond donor). DL-menthol and decanoic acid were heated up to 80 ˚C for 1 h under uniform stirring conditions of 350 rpm in an oil bath until a homogeneous liquid was formed. The liquid was then slowly cooled down for an additional two hours while stirring it constantly [S1].

S1.1 NMR studies of the dispersed phase with and without the lidocaine drug-loaded

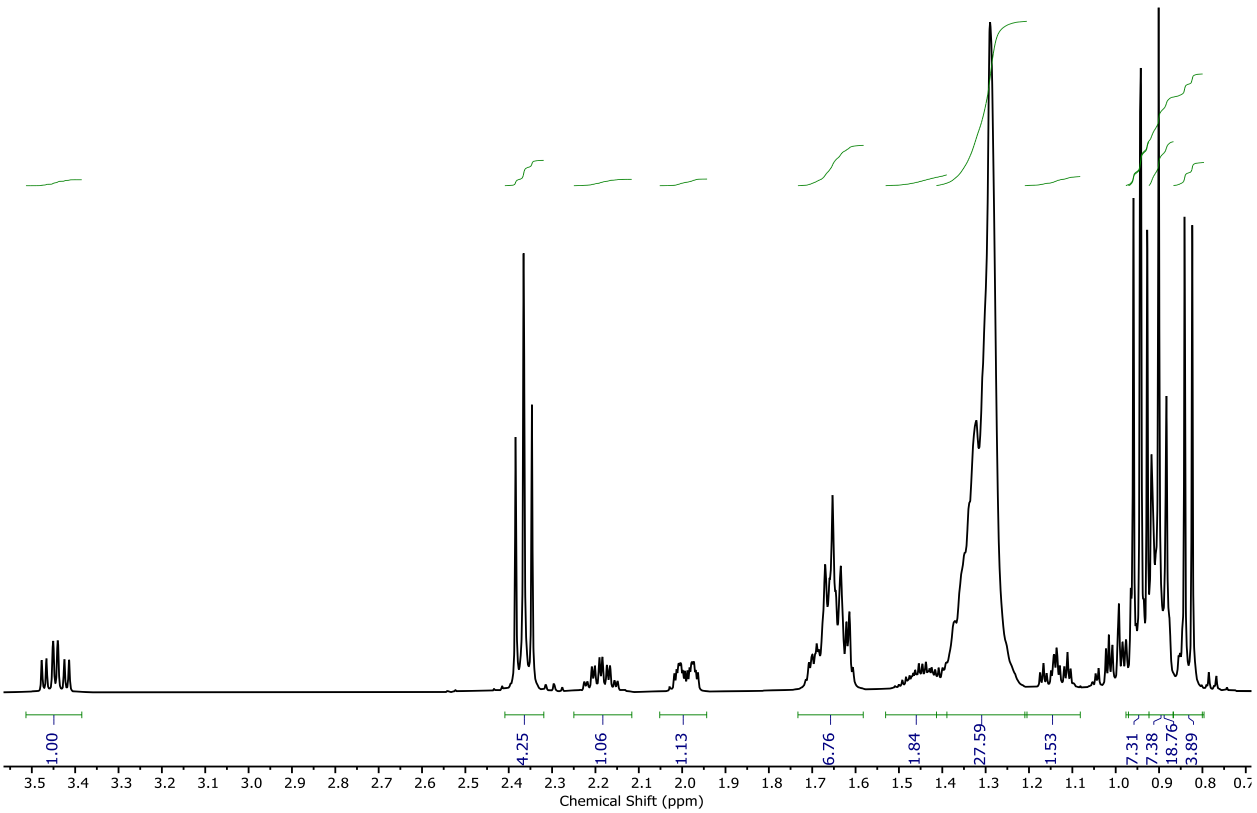

Fig. S1. NMR spectra of the DES comprising menthol and decanoic acid at 1:2 molar ratio

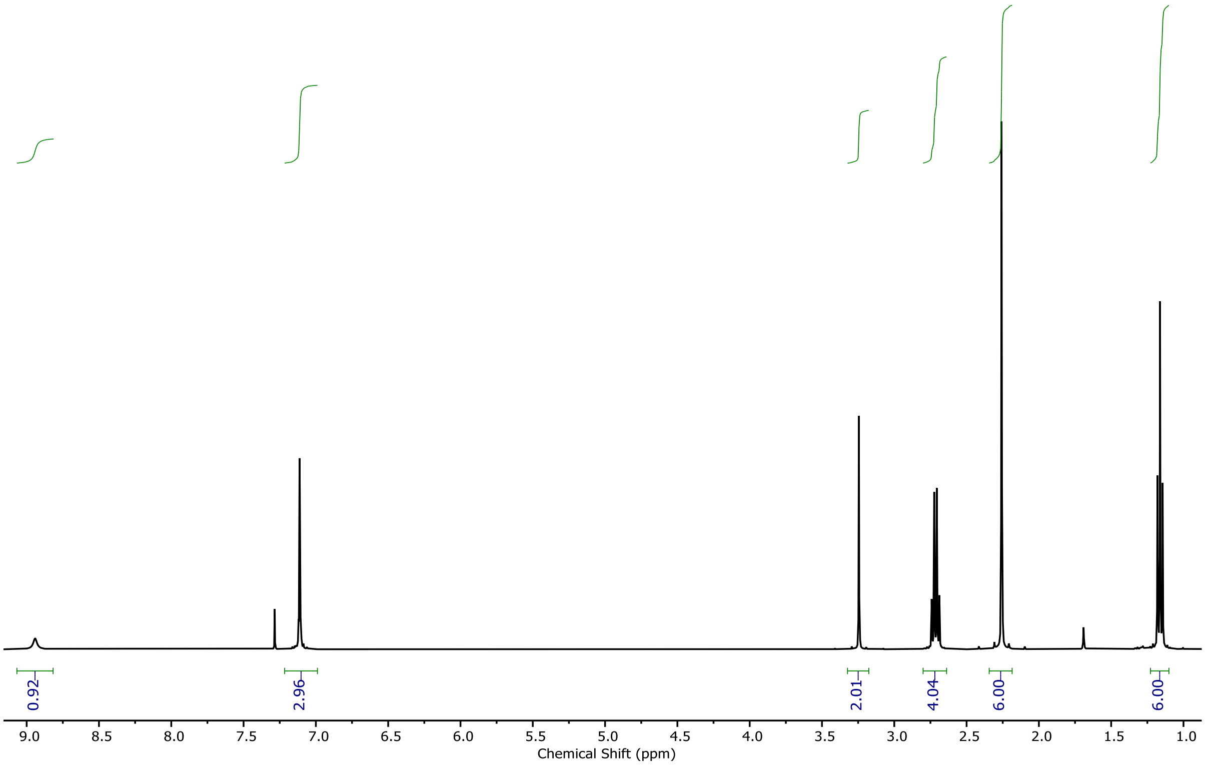

Fig. S2. NMR spectra of the pure lidocaine drug

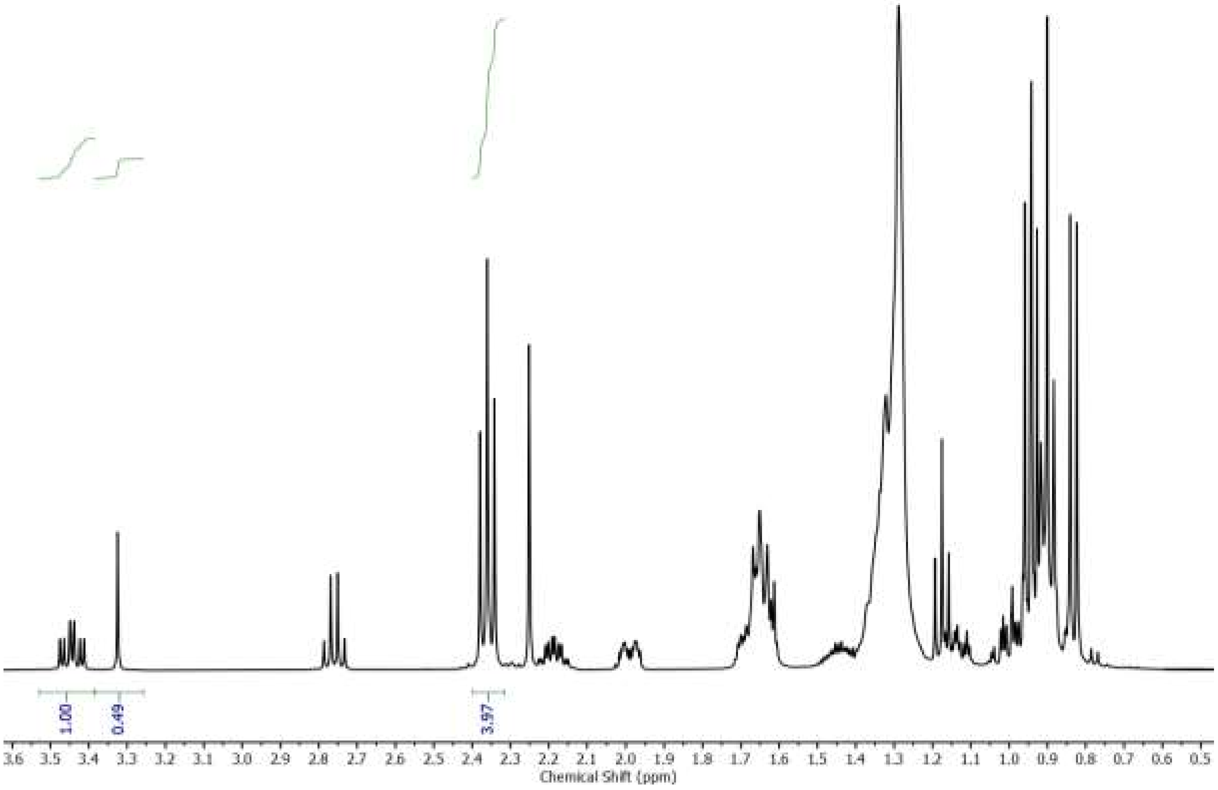

Fig. S3. NMR spectra with peaks of interest for 10 % (w/v) Lidocaine solubilized in the DES

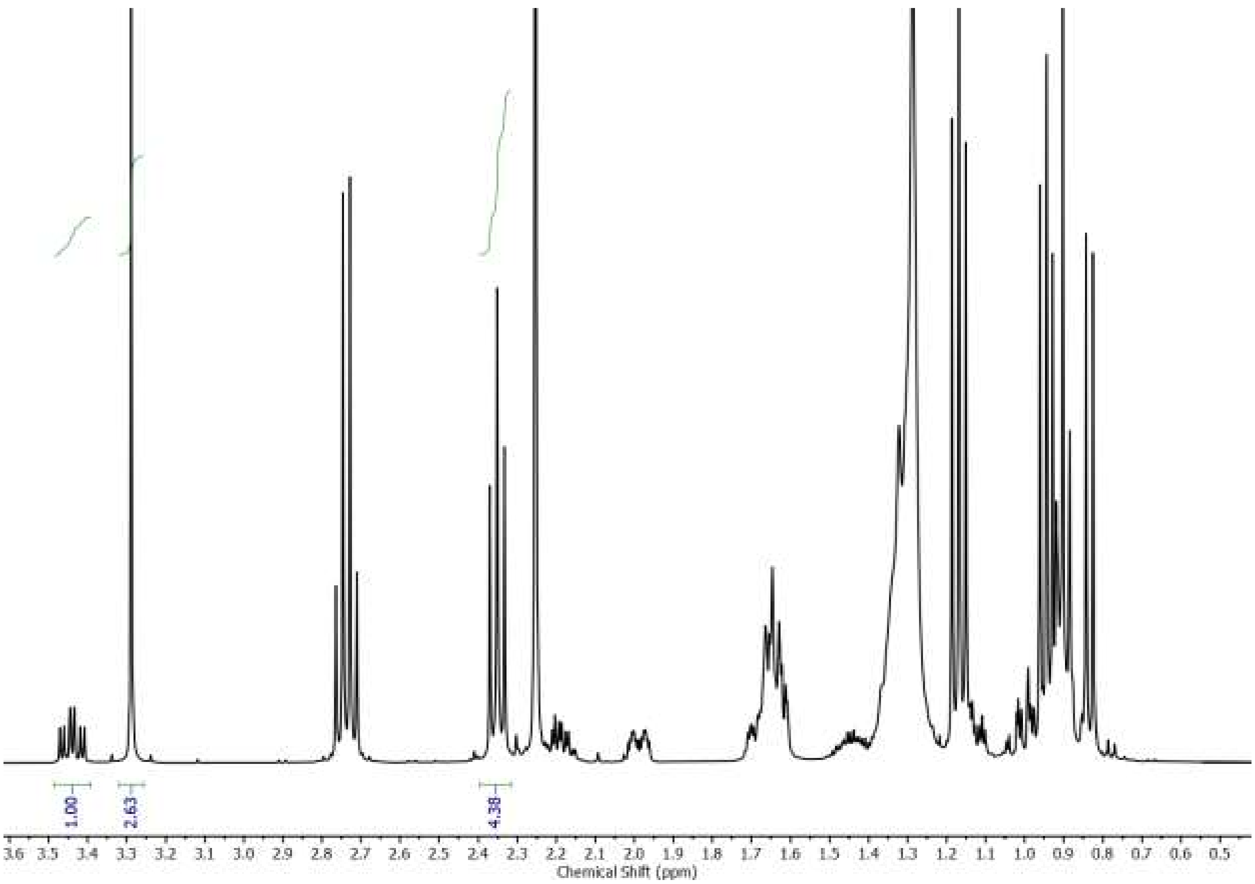

Fig. S4. NMR spectra with peaks of interest for 50 % (w/v) Lidocaine solubilized in the DES

S1.2 Interfacial tension measurements of the liquid phases used

The Drop Shape Analyzer (DSA 25B, Kruss GmbH, Germany) utilises the pendant drop method to measure the interfacial tension between the dispersed phase, with or without drug loaded in DES, and the continuous phase. A DS 1000/GT gas tight 500 µl glass syringe with a stainless steel dosing needle having 2 mm diameter was used for the formation of the bottom-up DES phase pendant drop within the aqueous phase. Interfacial tension values were obtained with the support of the software provided with the equipment from the fit of the pendant drop profile using the Laplace-Young equation. Measurements were performed at room temperature (23 ± 1 ˚C) and were repeated thrice. Averaged values were considered.

S2. Membrane emulsification studies

The schematic representation of the membrane emulsification set-up is depicted in Fig. S1. To produce small sized nanoemulsions, 2 mL of DES was dispersed through the metallic membrane

into 50 mL of continuous phase containing 2 % (w/w) Tween 20 in water, which was recirculated. The dispersed phase flowrate Q_{DP} [$m^3.s^{-1}$] was set on the automated syringe pump. The continuous phase cross-flow velocity v_c [$m.s^{-1}$] was calculated as follows:

$$v_c = \frac{4*Q_{CP}}{\pi*d_h^2} \quad (1)$$

where Q_{CP} [$m^3.s^{-1}$] is the continuous phase flow rate and d_h [m] is the hydraulic diameter of the flow channel on the continuous phase at the membrane module, which depends on the area of the cross sectional channel A [m^2] and perimeter of the channel P [m].

$$d_h = \frac{4A}{P} \quad [2]$$

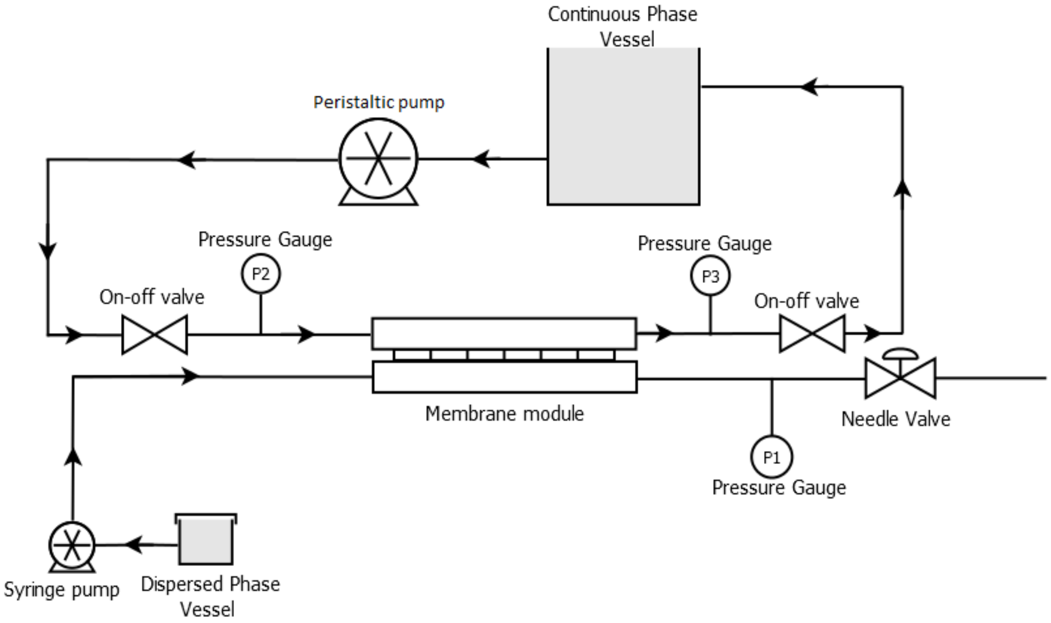

Fig. S5. Schematic representation of the membrane emulsification set-up used in this study.

Note: The active membrane area is 2.9×10^{-4} m^2 (as depicted in [S1]).

A 4 % (v/v) of DES was dispersed into 50 ml of the continuous phase comprising of 2 % (w/w) Tween 20 in aqueous solution. Three different samples of dispersed phase were studied: a) hydrophobic DES, b) 10 % (w/v) and c) 50 % (w/v) lidocaine solubilised in the DES. Two different dispersed phase flowrates Q_{dp} of 0.2 and 0.02 $ml.min^{-1}$ corresponding to 10 and 100 min of experimental time of emulsification, respectively, were chosen to evaluate their effect on the formulation of emulsion. Dynamic light scattering (DLS) was complemented by transmission

electron microscopy (TEM) studies to measure the emulsion droplet size and size distribution. Fig. 2 depicts the characterisation results of the optimised nanoemulsions.

S3. Characterisation of the emulsions produced

Dynamic Light Scattering was used to measure the droplet size distribution and polydispersity *PDI* of the emulsions by using a NanoZetaSizer particle size analyzer (Malvern instruments, Nano ZS, UK). The software used to collect and analyse the data was ZetaSizer Software 7.1 provided with the instrument. Experiments were triplicated at 25 °C.

To confirm the emulsion droplet sizes and size distribution, transmission electron microscopy (FEI Company, Japan) studies were carried out using a T20-FEI Tecnai thermionic microscope and operated at 200 keV. TEM samples were prepared by depositing 20 µl of the emulsion dispersed in milli-Q water onto a formvar coated copper TEM grid with negative stain of phosphotungstic acid and then dried for at least 2 hours.

Table S1 depicts the results of the membrane emulsification experiments for 3 different dispersed phases studied: a) hydrophobic DES, b) 10 % (w/v) lidocaine solubilised in the DES, and c) 50 % (w/v) lidocaine solubilised in the DES.

Table S1. Effect of dispersed phase flowrate on the Z_{avg} mean droplet size distribution by intensity and polydispersity index *'PDI'* of the DES-in-water nanoemulsions produced by membrane emulsification. **NOTE:** The continuous phase cross-flow velocity v_c [m.s^{-1}] is 0.32 m.s^{-1} for all the experiments.

Pore size of the active layer [µm]	Pore size of the bottom layer [µm]	Dispersed phase flowrate Q_{DP} [ml.min^{-1}]	Z_{avg} mean droplet size [nm]	Polydispersity index *PDI* [-]
**Dispersed phase: Hydrophobic DES**				
9	18	0.2	55.6 ± 2.7	0.38 ± 0.10
**9**	**18**	**0.02**	**58.7 ± 0.4**	**0.21 ± 0.02**
**Dispersed phase: 10 % (w/v) lidocaine solubilised in the DES**				
9	18	0.2	124.6 ± 1.7	0.28 ± 0.05
**9**	**18**	**0.02**	**91.1 ± 0.3**	**0.17 ± 0.05**
**Dispersed phase: 50 % (w/v) lidocaine solubilised in the DES**				

9	18	0.2	132.6 ± 2.4	0.40 ± 0.12
**9**	**18**	**0.02**	**52.1 ± 3.9**	**0.35 ± 0.08**

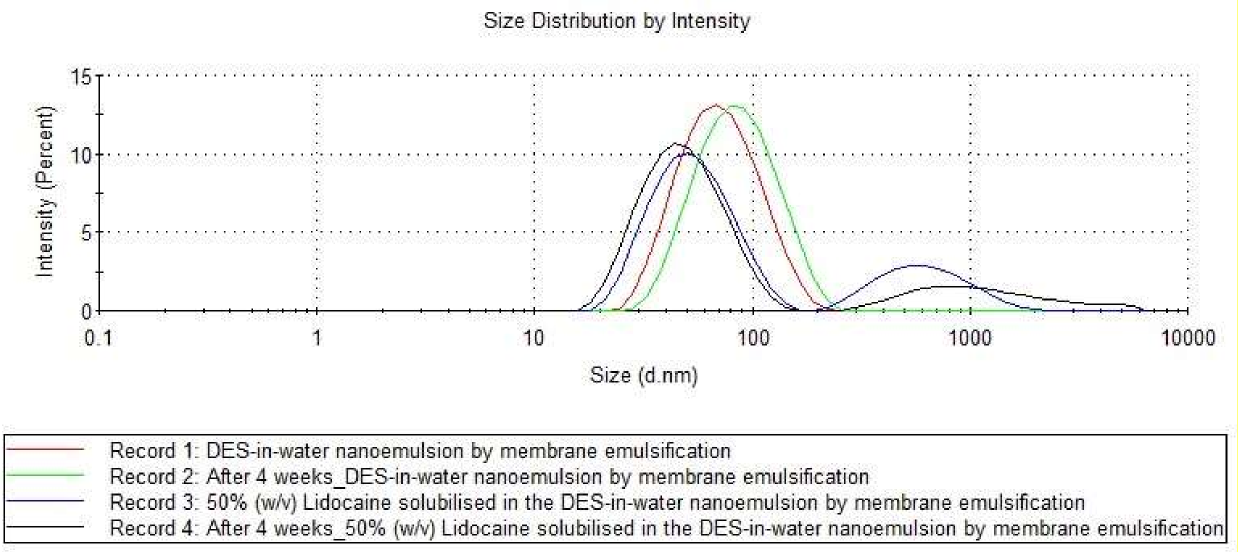

Fig. S6. Characterization of the long-term storage of nanoemulsions without and with the lidocaine encapsulated

S4. Ultra-Performance Liquid Chromatography (UPLC) studies to evaluate the release kinetics of the lidocaine drug

Lidocaine analysis was performed on a Waters ACQUITY system H-Class which consisted of a binary pump, an autosampler, a column thermostat and a photodiode array (PDA) detector. This system was coupled to a single quadrupole mass spectrometer with an electrospray ionization (ESI) ACQUITY QDa mass detector. Data acquisition and processing were performed using MASSLYNX software (Waters Corporation, USA).

Chromatographic separation was conducted using a CORTECS® UPLC C18 column (90 Å, 1.6 µm 2.1 x 100 mm, from WATERS) at 40 °C. The mobile phase consisted of an initial mixture of acetonitrile/water (90:10), containing 0.1 % formic acid. The initial flow rate was set at 0.25 ml.min^{-1}. Thereon, a gradient in the mobile phase was employed to improve the chromatographic resolution. Water content was increased for 4-5 mins until a 42 % acetonitrile was reached and, following this, the system reverted to its initial conditions.

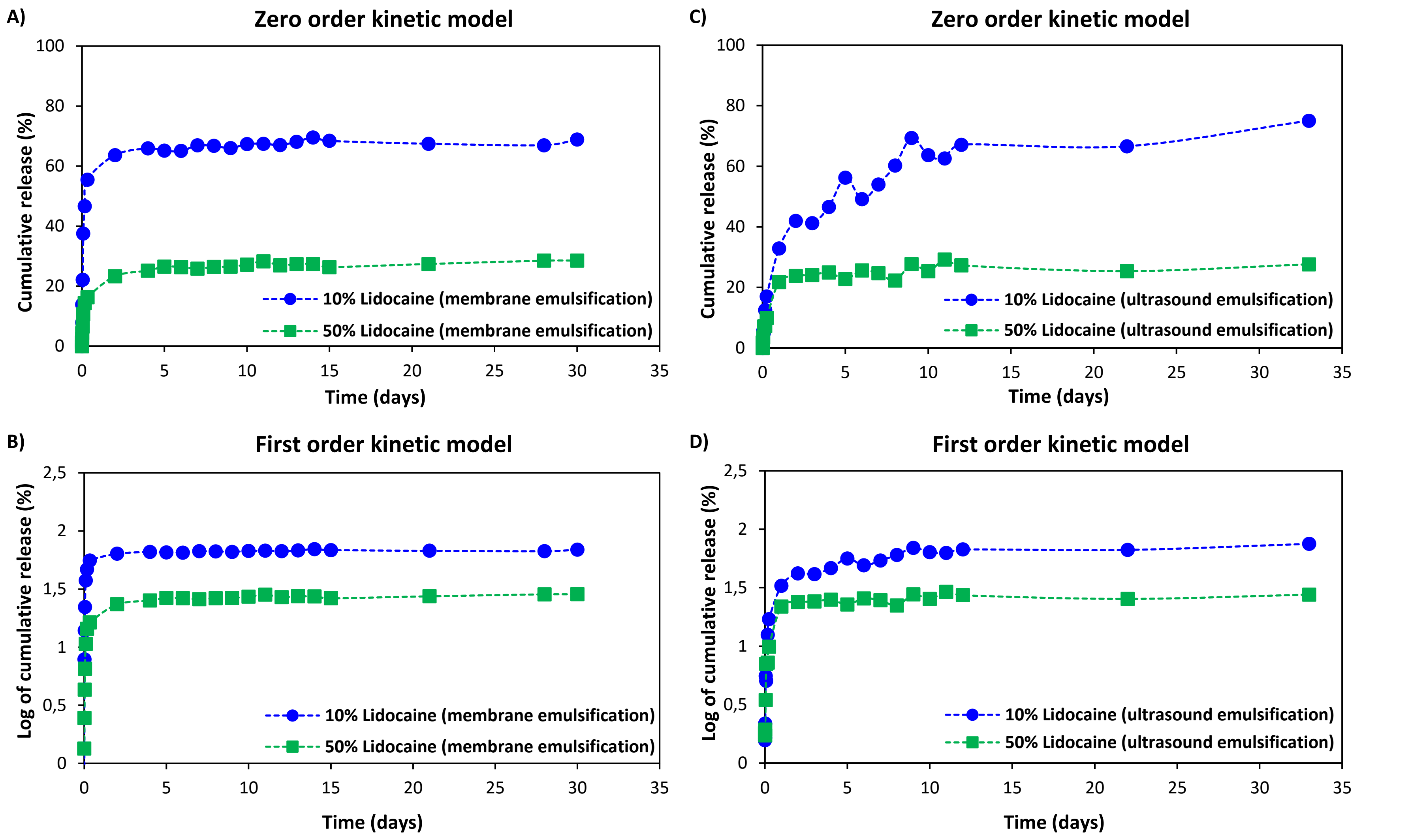

Fig. S7. Kinetic release studies of lidocaine drug loaded in DES-in-water nanoemulsions produced by two emulsification techniques. Membrane emulsification: A) Zero order kinetic model, B) First order kinetic model. Ultrasound emulsification: C) Zero order kinetic model, D) First order kinetic model.

An ACQUITY QDa mass detector was used to quantify lidocaine according to the most abundant ion, corresponding to a m/z ratio of 235.40 ($[M-H^+]^+$) and a PDA detector was employed to monitor absorbance from the drug at 254 nm during analysis time.

A calibration curve of lidocaine was obtained using commercial standards of lidocaine from 0.1 to 3.0 µg.ml^{-1} ($R^2 > 0.9979$). Both samples and standards were filtered using 0.22 µm Nylon filters before being injected into the UPLC system. Quantitative analysis was performed in triplicate. The profiles of the classical kinetic models such as zero order and first order models are depicted in Fig. S2. These two models consider a linear increase of a released component over a specified duration of time. The mathematical equations of the kinetic models used for evaluating the release of the lidocaine drug are presented in Table S2.

Table S2: Kinetic models used in the study to evaluate the kinetics of release of the drug [S2]

Sl. No.	Kinetic model	Mathematical equation	Parameters
1	Zero order	$Q_t = Q_0 + (k * t)$	○ Q_t is amount of drug released [mg]<br>○ Q_0 is the initial amount of drug in the solution [mg]<br>○ M_t/M_∞ is the fraction of drug released at time t<br>○ *k* is the release rate constants [mg.min^{-1}]<br>○ *n* is release exponent [-]
2	First order	$Log\ Q_t = Log\ Q_0 + (k * t)$	
3	Higuchi	$Q_t = Q_0 + (k * t^{0.5})$	
4	Korsemeyer-Peppas	$\frac{M_t}{M_\infty} = Q_0 + (k * t^n)$	

References

[S1] Syed, U. T.; Leonardo, I.; Lahoz, R.; Gaspar, F. B.; Huertas, R.; Crespo, M. T.; Arruebo, M.; Crespo, J. G.; Sebastian, V.; Brazinha, C. Microengineered Membranes for Sustainable Production of Hydrophobic Deep Eutectic Solvent-Based Nanoemulsions by Membrane Emulsification for Enhanced Antimicrobial Activity. ACS Sustainable Chem. Eng. 2020, 8(44), 16526-16536.

[S2] Albisa, A; Piacentini, E; Arruebo, M; Sebastian, V; Giorno, L. Sustainable production of drug-loaded particles by membrane emulsification. ACS Sustainable Chem. Eng. 2018, 13, 6(5), 6663-74.
